# Effect of acupuncture at the sphenopalatine ganglion for the treatment of moderate to severe seasonal allergic rhinitis: Study protocol for a three-armed randomized controlled trial

**DOI:** 10.3389/fmed.2022.904864

**Published:** 2022-09-06

**Authors:** Weiming Wang, Hui Chen, Ning Gao, Shudan Yu, Jiahua Liao, Shijie Wang, Ziqi Gao, Zhishun Liu

**Affiliations:** ^1^Department of Acupuncture and Moxibustion, China Academy of Chinese Medical Sciences Guang’anmen Hospital, Beijing, China; ^2^Department of Traditional Chinese Medicine, China Aerospace Science & Industry Corporation 731 Hospital, Beijing, China; ^3^Department of Otolaryngology, China Aerospace Science & Industry Corporation 731 Hospital, Beijing, China

**Keywords:** acupuncture, seasonal allergic rhinitis, sphenopalatine ganglion, randomized controlled trial, protocol

## Abstract

**Introduction:**

Seasonal allergic rhinitis (SAR) is a major health problem with a relatively high worldwide prevalence that severely limits the quality of life for sufferers. Acupuncture is widely used for SAR treatment in China; however, the evidence on the efficacy of acupuncture at the sphenopalatine ganglion (SPG) for SAR is inconclusive. Therefore, this study aims to investigate the efficacy and safety of acupuncture at the SPG acupoint for the treatment of SAR.

**Methods and analysis:**

A total of 120 participants with SAR will be recruited and randomly assigned to the acupuncture group, placebo acupuncture (PA) group, or rescue medication (RM) group with a 1:1:1 allocation ratio. Participants in the acupuncture group and PA group will receive 8 sessions of acupuncture stimulus at the SPG plus RM or 8 sessions of shallow needling at the SPG acupoint plus RM for 4 weeks with a 4-week follow-up in the first year and a 1-week follow-up in the second year. Participants in the RM group will only receive RM throughout the study. The primary outcome is the change from baseline in the average daily combined symptoms and medication score (CSMS) over weeks 1–4. All analysis will be based on an intention-to-treat principle. All statistical tests will be two-sided and a *p*-value < 0.05 will be considered to be statistically significant.

## Strengths and limitations of this study

► Acupuncture at the SPG might have a specific effect on the treatment of SAR. This study is the first randomized controlled trial that compares acupuncture at the SPG plus RM with shallow needling at SPG plus RM and only RM for participants suffering from SAR.

► This study was rigorously designed with strict inclusion and exclusion criteria, and the measurement of the primary outcome was recommended by the European Academy of Allergy and Clinical Immunology (EAACI), blinding participants, and outcome assessors.

► Due to the difficulty of inserting an acupuncture needle into the SPG, the acupuncturist must receive specialist training before the initiation of the trial.

► This is a single-center study that will only recruit patients with SAR in Asian populations, which might limit the generalizability of the study among other types of allergic rhinitis and other ethnic patients.

► The acupuncturists will not be blinded, which could potentially introduce bias in the results.

## Background

Allergic rhinitis (AR) is an immunoglobulin E-mediated inflammatory disease (1) that is caused by the hypersensitivity of the immune system to an allergen, which affects 100 million people in Europe (2) and 400 million people globally (3). Typical symptoms of AR include nasal congestion, rhinorrhea, itching, and sneezing (4), other common non-nasal symptoms include itchy eyes, tearing, and eye redness (5). Many patients with AR are susceptible to several comorbidities, such as asthma, rhinosinusitis, obstructive sleep apnea, and other related airway conditions (6). AR can result in impaired physical, emotional, and social functions, as well as poor quality of life (7), and therefore, has a substantial economic burden on society. The etiology of AR is multifactorial, which results primarily from a genetic predisposition, immunological response, and environmental pollutants (8). AR has traditionally been classified as seasonal allergic rhinitis (SAR) or perennial allergic rhinitis (PAR) depending on the causes and duration of symptoms (9).

In addition to avoiding allergens, current treatments for AR mainly include pharmacotherapy and immunotherapy (10). These treatments are effective to control and improve AR symptoms, but each treatment modality has unique challenges: it is impractical to eliminate all environmental allergens, pharmacotherapy (i.e., histamine antagonists) is often associated with adverse events, such as fatigue (11), and adherence with immunotherapy is often poor (12). Therefore, some patients with AR prefer complementary and alternative medicine to alleviate their symptoms, with approximately 20% receive acupuncture (13).

Acupuncture, which is one of the most studied Chinese medical techniques, involves stimulation of specific locations (acupoints) on the body, usually by the insertion of a fine needle (14). Many studies have reported the efficacy of acupuncture to treat AR (15–17), and a 2020 meta-analysis of 39 randomized clinical trials (RCTs) claimed that acupuncture methods were effective and safe for the treatment of AR (18). However, according to the updated practice parameters for rhinitis in 2020, the use of acupuncture for the treatment of AR was not recommended due to a lack of well-controlled studies (19).

The sphenopalatine ganglion (SPG), which is located under a thin (1–2 mm) layer of mucosa in the pterygopalatine fossa, consists of sensory fibers that innervate the nasopharynx, nasal cavity, and palate (20). Several studies reported the benefits of SPG stimulation for chronic cluster headaches (21), and acute ischemic stroke (22). Compared with traditional acupoints that are selected based on traditional meridian theory, acupuncture at the SPG (inserting a needle through the SPG acupoint near ST7, Xiaguan (23) to reach and directly stimulate the SPG) might help patients improve nasal symptoms immediately and improve their quality of life (24) by increasing sympathetic nerve excitability (25); however, the evidence is inconclusive.

This three-armed, randomized trial will investigate the efficacy and safety of acupuncture at the SPG for the treatment of SAR. Acupuncture at the SPG plus rescue medication (RM) might be superior to placebo acupuncture (PA) plus RM, and only RM for the treatment of SAR.

## Methods and design

### Study design

**FIGURE 1:**
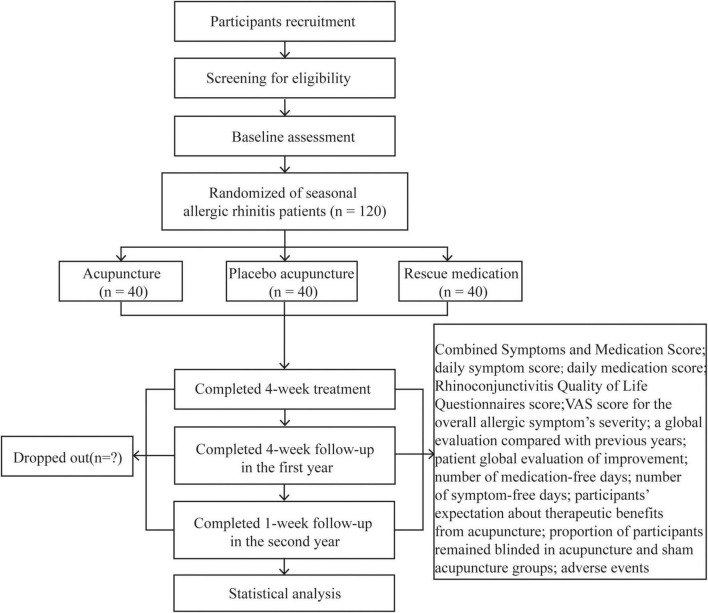
Study flow diagram.

**FIGURE 2:**
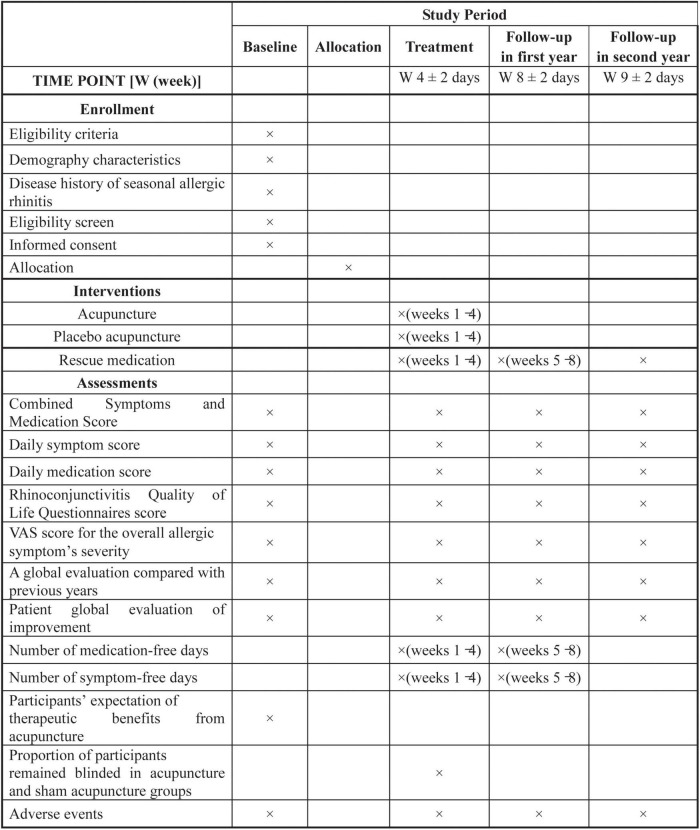
Study schedule.

This is a parallel-design, three-armed, patient-assessor blinded randomized (1:1:1) controlled trial. This protocol has been developed according to the standard protocol items included in the *Recommendations for Interventional Trials* (26) and the *Standards for Reporting Interventions in Clinical Trials of Acupuncture* (27) guidelines. The trial flow diagram and treatment schedule are shown in Figures 1 and 2.

### Study setting and recruitment

This trial will be carried out at No. 731 Hospital of China Aerospace Science and Industry Corporation from May 2021 to August 2023. A total of 120 participants will be recruited. The trial duration will be 10 weeks: 1 week baseline (run-in phase), 4 weeks treatment, 4 weeks follow-up in the first year, and the first week following symptom onset in the second year. At baseline, participants would not take any anti-histamines and will record their SARs symptoms in a daily participant diary.

### Randomization and blinding

Participants who agreed to randomization will be allocated to the acupuncture, PA, or RM groups in a 1:1:1 ratio that uses a fixed block size of 6. The randomization number of the allocation sequence will be generated using PROC PLAN of SAS software, version 9.4 (SAS Institute Inc., Cary, NC, United States). An independent researcher will prepare consecutively numbered, sealed, opaque envelopes that contain the information about group allocation. These envelopes will be consecutively opened by a research coordinator that is not involved in recruitment, therapy, and outcome assessments immediately after the baseline assessments.

Because of the two different acupuncture techniques that will be used in this trial, the acupuncturist will know which group each participant is in. However, the participants in the acupuncture and PA groups, outcome evaluators, and statisticians will be blinded to the group allocation throughout the trial. To ensure blinding, all researchers will receive the same training before the trial, and each participant will be treated in a separate room. Participants in the RM group will not be blinded.

### Participants

The participants will have previously been diagnosed with SAR by a lung physician or allergist, according to the Allergic Rhinitis and Its Impact on Asthma (ARIA) criteria (28). Participants will be recruited during the pollen season, which is defined as the period with pollen levels ≥ 20 grains/m (3, 29). According to a previous study, the pollen season annually in Beijing was set from 17 March to the end of October (30). To ensure that participants are recruited within pollen season from baseline to the end of the 4 weeks follow-up period in the first year, patients would not be involved in the same year, if pollen season is over in less than 9 weeks. Participants will be eligible if they meet all the inclusion criteria and have none of the exclusion criteria. The last participant is expected to complete treatment in September 2021.

### Inclusion criteria

1. Aged ≥ 18 years and ≤ 75 years.
2. A history of moderate to severe SAR symptoms [visual analog scale (VAS) > 50 mm, range from 0 cm (not at all troublesome) to 100 mm (extremely bothersome)] (31) for > 4 days per weeks, and > 4 consecutive weeks with ≥ 2 years duration.
3. Participants’ SAR symptoms severity scores at baseline > 50 mm for at least 4 consecutive days at baseline.
4. Positive skin prick test response, defined as wheal diameter greater than or equal to 3 mm, to grass and birch pollen (rather than dust mite or mold), or a serum-specific IgE test, or both.
5. Ability to complete the medical information form and sign a written informed consent.

### Exclusion criteria

1. A history or current evidence of PAR, acute sinusitis, allergic asthma, pneumonia, autoimmune disorders, or severe chronic inflammatory diseases.
2. A history of nasal rhinopolypus or abnormalities.
3. Taking antihistamines, anticholinergics, corticosteroids, decongestants, or antibiotics 1 month before starting the study.
4. A history of systemically administered corticosteroids within 6 months or specific immunotherapy, or allergy desensitization therapy 1 year before enrollment.
5. Serious uncontrolled blood coagulation disorder, cardiovascular disorder, severe hepatic or renal insufficiency, or a mental disorder.
6. Pregnancy or planning for pregnancy;
7. Known allergy, or contraindication to RM or related drugs.
8. Known phobia to acupuncture or have received acupuncture treatment, or SPG stimulation, or other complementary and alternative medicine within 1 month of enrollment.

### Interventions

#### Acupuncture group

**FIGURE 3:**
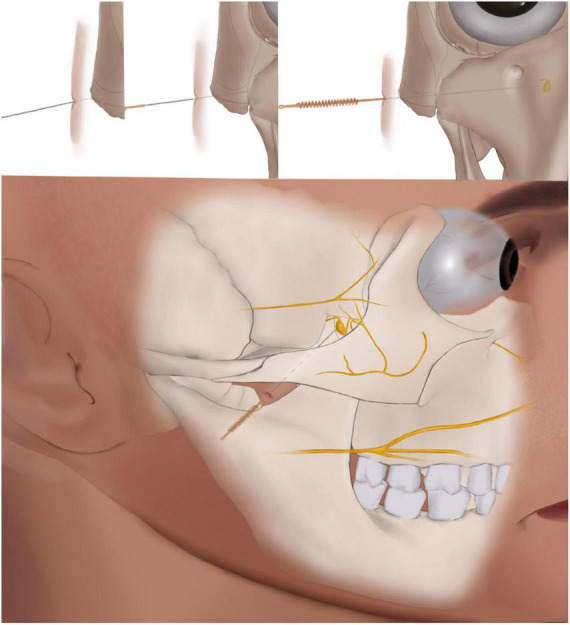
Schematic diagram of acupuncture.

The acupuncture regimen was determined based on previous reports (23, 24, 32). The licensed acupuncturists have > 5 years of practical experience. Before the study, the acupuncturists will receive special training in the SPG stimulation technique and will perform the technique clinically. The SPG acupoint is located under the zygomatic arch between the coronoid process and mandibular condyle (24). Sterile single-use stainless steel needles (0.35 mm × 55 mm; YiDaiFu brand, Suzhou Tianyi Acupuncture Instrument Co., Ltd., Suzhou, China) will be used. Participants will be in the lateral position and the acupoints area will be sterilized with 75% alcohol. To stimulate the SPG, the needles will be inserted into the medial superior anterior direction to a depth of approximately 55 mm (33), until the participants report a special (deqi) sensation that radiates toward the nose or the upper teeth (Figure 3). Then, the needle will be withdrawn slightly. The needles will be retained for 30 min and stimulated 3 times during the needling period.

#### Placebo acupuncture group

The acupuncture procedure is similar to that of the acupuncture group. After sterilizing the skin, the 0.35 × 25 mm disposable needle will be vertically inserted at the SPG acupoint approximately 3–5 mm. The needles will also be retained for 30 min, yet no needle manipulation would be carried out to avoid the deqi (unique) response.

Acupuncture is administrated unilaterally. The SPG acupoint will be stimulated alternatively in each session. After randomization, participants receive treatment twice per week for eight sessions for four consecutive weeks. All participants will be treated separately to prevent communication and will be advised to avoid allergens during the trial.

#### Rescue medication group

Participants in the RM group will not receive acupuncture treatment during the study period. They could use the RM described in the following section. They have the option of 4 weeks (≤ 8 sessions) of acupuncture free of charge at the end of the follow-up period.

#### Rescue medication

The following RM; non-sedative H1 antihistamines, intranasal, or oral corticosteroids are permitted in each group based on a standardized approach (34), only when participants feel that their symptoms are intolerable. Administration for prophylactic purposes is prohibited. Additional medications, such as leukotriene receptor antagonists, anticholinergic agents, α-adrenergic agonists, allergen immunotherapy, nasal ipratropium, decongestants, or any form of alternative therapy are not allowed at any time during the study period. The daily medication score (dMS) will be recorded every day in case report forms (CRFs) based on the following scores: 0 = no RM; 1 = use of oral, or topical non-sedative H1 antihistamines, or both (e.g., Clarityne or Patanol); 2 = use of intranasal corticosteroids (Rhinocort) with or without H1 antihistamines; and 3 = use of oral corticosteroids (Prednisone) with or without intranasal corticosteroids, with or without H1 antihistamines (34). When a participant takes ≥ 2 rescue medications, the higher score will be retained for the corresponding day.

### Outcome measures

#### Primary outcome

The primary outcome is the change from baseline in the average daily combined symptoms and medication score (CSMS) over weeks 1–4, which measures the symptoms of AR and the use of RM. It has been widely used in previous studies and is recommended by the EAACI (34). The average daily CSMS is the sum of the daily symptom score (dSS) plus dMS. The dSS contains a 6-item scale that refers to nasal symptoms (4 items) and ocular symptoms (2 items), and each item is scored using a Likert scale of 0–3. The dSS is calculated as a mean of all entered dSS divided by the number of individual symptoms (range 0–3). The dMS is calculated as an average of the daily symptom relief medication score, with a range of 0–3. Therefore, the potential CSMS score could be from 0 to 6, with high scores indicating more severe nasal symptoms. In addition, the changes from baseline over weeks 5–8 during the first year and the first week following symptoms onset in the second year will be assessed.

#### Secondary outcomes

The secondary outcomes are:

1. Change in the average dSS and dMS from baseline over weeks 1–4, weeks 5–8 in the first year, and the first week following symptoms onset in the second year.
2. The proportion of participants with a minimum of 23% improvement in the average daily CSMS from baseline over weeks 1–4, weeks 5–8 in the first year, and at the first week following symptoms onset in the second year. Based on the previous data, a difference of 23% (35) in the average daily CSMS was chosen to demonstrate a minimum clinically significant difference.
3. A change in the Rhinoconjunctivitis Quality of Life Questionnaires (RQLQ) (36) total score and subscale scores from baseline to the end of weeks 4 and 8 in the first year and the first week following symptoms onset in the second year. The RQLQ is a well-established and validated questionnaire that consists of 28 questions that cover 7 domains: (1) sleep (3 items); (2) practical problems (3 items); (3) non-nasal and eye symptoms (7 items); (4) nasal symptoms (4 items); (5) eye symptoms (4 items); (6) activities that have been limited by nose or eye symptoms (3 items); and (7) and emotional function (4 items). Each item was evaluated on a 7-point rating scale from 0 (no impairment) to 6 (severe impairment) (36) during the previous week. The analysis of the RQLQ total score is the average of the answers to the 28 items and the subscale scores are the average of the answers in those subscales. The total score or the subscale score is between 0 and 6, with high values indicating the lower disease-specific quality of life. Changes in scores by ≥ 0.5 were considered clinically significant (37). In this study, the validated Chinese version of RQLQ is used (38).
4. A Change in the VAS score for the overall allergic symptom’s severity from baseline to the end of weeks 4 and 8 in the first year and the first week following symptoms onset in the second year. Patients will be asked to grade the severity of allergic symptoms using the self-rated 0–100 mm VAS (0 = no symptoms and 100 = worst-ever symptoms, in 1-point increments). The VAS is a reliable and valid tool to quantitatively evaluate AR severity (31).
5. A global evaluation will compare previous years by each participant at the end of weeks 4 and 8 in the first year and the first week following symptoms onset in the second year. Each participant will be asked the following question: Compared with your symptoms in previous grass pollen seasons, how have you felt overall in this grass pollen season? (select only one). The possible answers are coded follows:1 = very much better; 2 = much better; 3 = a little better; 4 = no change; 5 = a little worse; 6 = much worse; and 7 = very much worse.
6. Patient global evaluation of improvement at the end of weeks 4 and 8 in the first year and the first week following symptoms onset in the second year. Patient global evaluation of improvement will be rated by the participants using a 7-point Likert scale with the following options: 1 = very much better; 2 = much better; 3 = a little better; 4 = no change; 5 = a little worse; 6 = much worse; and 7 = very much worse, at each study visit.
7. The average weekly number of medication-free days during weeks 1–4 and weeks 5–8 in the first year.
8. The average weekly number of symptom-free days during weeks 1–4 and weeks 5–8 in the first year.
9. Participants expectation about the therapeutic benefits of acupuncture at baseline. At baseline, participants in the acupuncture and PA groups will be asked the following question: How helpful you believe the acupuncture modality you received would be for your AR. Participants will be instructed to choose one of the given answers: (1) very helpful; (2) moderately helpful; (3) slightly helpful; (4) not helpful; and (5) unclear.
10. The participants remained blinded to the treatment arm in the acupuncture and PA groups. Five minutes after the end of the final treatment in week 4, each participant in the acupuncture and PA groups will be asked the following question: Which treatment do you think you received (acupuncture or PA). Participants will be allowed to choose only one answer, acupuncture, “PA,” or unclear. Before the question, participants will be told that they might have received acupuncture with a deeper insertion or PA with shallow penetration.

### Safety assessment

Any potential adverse events (AEs) will be monitored and documented in the CRFs within 24 h of their occurrence during treatment and the follow-up period. Based on their potential association with acupuncture, AEs will be categorized as acupuncture-associated AEs (e.g., subcutaneous hemorrhage, dizziness, fainting, serious pain, local infection, and localized hematoma), and non-treatment-related AEs. Any serious adverse events, for example, an event that is life-threatening, or requires hospitalization, or results in death, hospitalization, or significant disability will be reported immediately to the study principal investigator and ethics committee. The ethics committee will decide whether to suspend the trial if required, and the statistician will break the blinding. Any participant that suffers an SAE will be withdrawn from the study.

### Sample size calculation

This trial hypothesizes that 4 weeks of acupuncture treatment plus RM could be superior for the improvement of the average daily CSMS over PA plus RM and only RM. Based on previous studies (15, 16), the differences in total nasal symptom score that changed at week 4 and the RM score that changed at week 8 between acupuncture and PA were 1.00 and 1.1, respectively. The between-group difference of 1.65 (1 point change in symptom score and 0.55 point change in RM score) with a standard deviation of 2.0 in the improvement of total CSMS could be detected at week 4 in the first pollen season period. Assuming an α = 0.05 level of significance (two-sided test), 90% power, and a 20% drop-out rate, 120 patients (40 in each group) will be considered.

### Statistical analysis

The null hypothesis is that the change from baseline in the average daily CSMS over weeks 1–4 in the first year could be the same in the acupuncture, the PA group and RM groups. Data will be presented as means with standard deviations, or medians with interquartile ranges for continuous variables, and frequencies (number of cases) or relative frequencies (%) for categorical variables. The repeated measure of the analysis of variance (ANOVA) will be performed for normally distributed variables, and a non-parametrical Kruskal–Wallis test will be used for non-normally distributed variables. The categorical variables will be compared using the Chi-squared (χ^2^) test. A two-tailed *p*-value < 0.05 will be considered statistically significant. All analyses will be conducted based on an intention-to-treat approach with all randomized participants included using SPSS software V.20.0 (IBM SPSS Statistics; IBM Corp, Somers, NY). Missing primary outcome data will be handled by multiple imputation techniques according to the missing at random assumption. Missing data will not be imputed for secondary outcomes.

### Quality control

To guarantee the quality of the trial, all participating staff will receive the same study training before the study starts. The training includes the aim of the trial, case screening and recruitment, intervention protocols, outcome measures, and data processing. The licensed acupuncturists have ≥ 5 years of clinical acupuncture practice and will receive special training in the SPG stimulation technique. The principal investigator has overall responsibility for the trial and is supported by a research coordinator for the CRFs review, data entry verification and storage, and quality control checks. Dropouts and withdrawals from the study will be documented during the trial. All paper data will be stored in a fire-proof safe, and all-electric data saved on a secure server within No. 731 Hospital of China Aerospace Science and Industry Corporation.

### Patient and public involvement

The research question was first proposed by a patient that failed the conventional acupuncture treatment. Patients were not involved in the development or implementation of this study. The results of this study will be communicated to all participants after completion of the study upon their request.

## Discussion

AR is a global health problem, which severely impairs the sufferers quality of life (7). Previously, acupuncture has been widely used to alleviate associated symptoms that are induced by AR. However, there is an ongoing debate on the effect of acupuncture on SAR. To the best of our knowledge, this is the first trial that aims to evaluate the clinical efficacy of acupuncture at the SPG for SAR that uses the CSMS recommended by the EAACI. The results of this trial could help to determine the effect of acupuncture at the SPG to improve SAR symptoms and reduce RM.

Although the SPG is relatively small and varies in size between individuals, it is possible to reach the SPG by inserting a needle through the SPG acupoint (33). A previous study found that acupuncture at the SPG led to significant improvement in nasal ventilation and nasal patency, and increased sympathetic nerve excitability (25) in healthy volunteers. In addition, one pilot study revealed the effect of acupuncture at the SPG acupoint for the prevention of PAR development (24). In this study, the effect of acupuncture at the SPG for SAR will be determined. Due to ethical considerations, the participants will be allowed to use relief medication, and therefore, the total CSMS was selected as the primary outcome measure as recommended by the EAACI (34). The CSMS is easy to understand and is an analysis of the daily burden of the disease, which equally combines symptom scores and medication scores.

This prospective study is a registered, concealed-allocation, three-armed, randomized controlled trial. The strengths of the trial include strict inclusion and exclusion criteria, the measurement of the primary outcome as recommended by the EAACI, evaluation of the participant’s expectation for acupuncture, blinding of the participants and outcome assessors, and is analyses based on the principle of intent-to-treat. In addition, this trial has several limitations. First, this is a single-center study in an Asian population, which might limit the generalizability of the study among other ethnic patients. Second, only patients with SAR were included, and therefore, the result might not apply to other types of AR (i.e., PAR). Third, the acupuncturists cannot be blinded due to the nature of acupuncture, which might bias the results of this study.

## Ethics statement

This study will be conducted in compliance with the principles of the Declaration of Helsinki 2008. This study was approved by the Medical Ethics Committee of No. 731 Hospital of China Aerospace Science and Industry Corporation (approval No: 2021-0102-01) on March 12, 2021. The registration number provided by ClinicalTrials.gov is NCT04815668. All investigators will adhere to a strict confidentiality regulation before, during, and after the trial. All participants will be asked to voluntarily sign the informed consent form if they agree to participate and will be reminded that they can withdraw from the study at any time without giving a reason. They also can be discontinued from the trial by the investigator if based upon his clinical judgment, continuation in the trial is deemed inappropriate. Any modifications to the protocol that might impact the conduct of this study will be submitted to the ethics committee and will be updated in the clinical trial registry. After completing data analysis, the results of this study will be published in an international peer-reviewed medical journal and will be disseminated via national conferences and scientific meetings.

## Author contributions

WW, ZL, and HC contributed to the conception and design of the study. HC, JL, SW, and NG will be responsible for clinical recruitment, intervention, data collection, and outcome assessment, respectively. ZG will be responsible for statistical analysis. This manuscript was drafted by WW and revised by SY and ZL. All authors read and approved the final manuscript.

## References

[1] Seidman MD Gurgel RK Lin SY Schwartz SR Baroody FM Bonner JR Clinical practice guideline: allergic rhinitis executive summary. *Otolaryngol Head Neck Surg.* (2015) 152:197–206. 10.1177/0194599814562166 25645524

[2] European Academy of Allergy and Clinical Immunology. *Advocacy Manifesto: Tackling the Allergy Crisis in Europe-Concerted Policy Action Needed.* Brussels: EAACI – EU Liaison Office (2015).

[3] Pawanker R Canonica G Holgate S editors. *White Book on Allergy: Update.* (Vol. 2013). Milwaukee, WI: World Allergy Organization (2013).

[4] Dykewicz MS Wallace DV Baroody F Bernstein J Craig T Finegold I Treatment of seasonal allergic rhinitis: an evidence-based focused 2017 guideline update. *Ann Allergy Asthma Immunol.* (2017) 119:489–511. 10.1016/j.anai.2017.08.012 29103802

[5] Wallace DV Dykewicz MS Bernstein DI BlessingMoore J Cox L Khan DA The diagnosis and management of rhinitis: an updated practice parameter. *J Allergy ClinImmunol.* (2008) 122(Suppl. 2):S1–84. 10.1016/j.jaci.2008.06.003 18662584

[6] Hadley JA Derebery MJ Marple BF. Comorbidities and allergic rhinitis: not just a runny nose. *J FamPract.* (2012) 61(Suppl. 2):S11–5. 22312619

[7] Horak F. Clinical advantages of dual activity in allergic rhinitis. *Allergy.* (2000) 55(Suppl. 64):34–9. 10.1034/j.1398-9995.2000.00805.x 11291779

[8] Lim R Fedulov AV Kobzik L. Maternal stress during pregnancy increases neonatal allergy susceptibility: role of glucocorticoids. *Am J Physiol Lung Cell Mol Physiol.* (2014) 307:L141–8. 10.1152/ajplung.00250.2013 24838749 PMC4101791

[9] Us Department of Health and Human Services. *Allergic Rhinitis: Developing Drug Products for Treatment Guidance for Industry: Draft Guidelines 2016.* Rockville, MD: Federal Register (2020).

[10] May JR Dolen WK. Management of allergic rhinitis: a review for the community pharmacist. *ClinTher.* (2017) 39:2410–9. 10.1016/j.clinthera.2017.10.006 29079387

[11] Yanai K Rogala B Chugh K Paraskakis E Pampura AN Boev R. Safety considerations in the management of allergic diseases: focus on antihistamines. *Curr Med Res Opin.* (2012) 28:623–42. 10.1185/03007995.2012.672405 22455874

[12] Meltzer EO Blaiss MS Naclerio RM Stoloff SW Derebery MJ Nelson HS Burden of allergic rhinitis: allergies in America, Latin America, and Asia-Pacific adult surveys. *Allergy Asthma Proc.* (2012) 33(Suppl. 1):S113–41. 10.2500/aap.2012.33.3603 22981425

[13] Krouse JH Krouse HJ. Patient use of traditional and complementary therapies in treating rhinosinusitis before consulting an otolaryngologist. *Laryngoscope.* (1999) 109:1223–7. 10.1097/00005537-199908000-00007 10443823

[14] Vickers A Wilson P Kleijnen J. Acupuncture. *QualSaf Health Care.* (2002) 11:92–7. 10.1136/qhc.11.1.92 12078381 PMC1743552

[15] Choi SM Park JE Li SS Jung H Zi M Kim TH A multicenter, randomized, controlled trial testing the effects of acupuncture on allergic rhinitis. *Allergy.* (2013) 68:365–74. 10.1111/all.12053 23253122

[16] Brinkhaus B Ortiz M Witt CM Roll S Linde K Pfab F Acupuncture in patients with seasonal allergic rhinitis: a randomized trial. *Ann Intern Med.* (2013) 158:225–34. 10.7326/0003-4819-158-4-201302190-00002 23420231

[17] McDonald JL Smith PK Smith CA Xue CC Golianu B Cripps AW Effect of acupuncture on house dust mite specific IgE, substance P, and symptoms in persistent allergic rhinitis. *Ann Allergy Asthma Immunol.* (2016) 116:497–505. 10.1016/j.anai.2016.04.002 27156748

[18] Yin Z Geng G Xu G Zhao L Liang F. Acupuncture methods for allergic rhinitis: a systematic review and bayesian meta-analysis of randomized controlled trials. *Chin Med.* (2020) 15:109. 10.1186/s13020-020-00389-9 33062045 PMC7552548

[19] Dykewicz MS Wallace DV Amrol DJ Baroody FM Bernstein JA Craig TJ Rhinitis 2020: a practice parameter update. *J Allergy ClinImmunol.* (2020) 146:721–67. 10.1016/j.jaci.2020.07.007 32707227

[20] Robbins MS Robertson CE Kaplan E Ailani J Charleston IVL Kuruvilla D The sphenopalatine ganglion: anatomy, pathophysiology, and therapeutic targeting in headache. *Headache.* (2016) 56:240–58. 10.1111/head.12729 26615983

[21] Goadsby PJ Sahai-Srivastava S Kezirian EJ Calhoun AH Matthews DC McAllister PJ Safety and efficacy of sphenopalatine ganglion stimulation for chronic cluster headache: a double-blind, randomised controlled trial. *Lancet Neurol.* (2019) 18:1081–90. 10.1016/S1474-4422(19)30322-9 31701891

[22] Bornstein NM Saver JL Diener HC Gorelick PB Shuaib A Solberg Y An injectable implant to stimulate the sphenopalatine ganglion for treatment of acute ischaemic stroke up to 24 h from onset (ImpACT-24B): an international, randomised, double-blind, sham-controlled, pivotal trial. *Lancet.* (2019) 394:219–29. 10.1016/S0140-6736(19)31192-4 31133406

[23] Li XW Tian ZP. A preliminary summary of the treatment on rhinitis puncturing sphenopalatine ganglion. *Beijing Chin Med.* (1990) 9:36–8.

[24] Mi J Chen X Lin X Guo J Chen H Wei L Treatment of persistent allergic rhinitis via acupuncture at the sphenopalatine acupoint: a randomized controlled trial. *Trials.* (2018) 19:28. 10.1186/s13063-017-2339-z 29325594 PMC5765676

[25] Wang K Chen L Wang Y Wang C Zhang L. Sphenopalatine ganglion acupuncture improves nasal ventilation and modulates autonomic nervous activity in healthy volunteers: a randomized controlled study. *Sci Rep.* (2016) 6:29947. 10.1038/srep29947 27425415 PMC4947913

[26] Chan AW Tetzlaff JM Gøtzsche PC Altman DG Mann H Berlin JA SPIRIT 2013 explanation and elaboration: guidance for protocols of clinical trials. *BMJ.* (2013) 346:e7586. 10.1136/bmj.e7586 23303884 PMC3541470

[27] MacPherson H Altman DG Hammerschlag R Youping L Taixiang W White A Revised STandards for reporting interventions in clinical trials of acupuncture (STRICTA): extending the CONSORT statement. *Acupunct Med.* (2010) 28:83–93. 10.1136/aim.2009.001370 20615861 PMC3002761

[28] Bousquet J Khaltaev N Cruz AA Denburg J Fokkens WJ Togias A Allergic rhinitis and its impact on asthma (ARIA) 2008 update (in collaboration with the World Health Organization, GA(2)LEN and Aller- Gen). *Allergy.* (2008) 63(Suppl. 86):8–160. 18331513 10.1111/j.1398-9995.2007.01620.x

[29] Rondón C Blanca-López N Campo P Mayorga C Jurado-Escobar R Torres MJ Specific immunotherapy in local allergic rhinitis: a randomized, double-blind placebo-controlled trial with *Phleum pratense* subcutaneous allergen immunotherapy. *Allergy.* (2018) 73:905–15. 10.1111/all.13350 29168570

[30] Meng L Wang XK Ouyang ZY Ren YF Lu F. Seasonal dynamics of airborne pollen in Beijing Urban area. *Acta Ecol Sin.* (2013) 33:2381–7. 10.5846/stxb201204100502

[31] Bousquet PJ Combescure C Neukirch F Klossek JM Mechin H Daures JP Visual analog scales can assess the severity of rhinitis graded according to ARIA guidelines. *Allergy.* (2007) 62:367–72. 10.1111/j.1398-9995.2006.01276.x 17362246

[32] Zhang WG Wang ZF Wu BH. Acupuncture with warmed needle in treating 30 cases of knee osteoarthritis. *J Fujian Coll TCM.* (2007) 17:39–41.

[33] Zhang L Fang DL Jiang DW Gao Y Shi DZ. Can the sphenopalatine ganglion be reached by an acupuncture needle? *Acupunct Med.* (2017) 35:153–5. 10.1136/acupmed-2016-011216 28043941 PMC5466912

[34] Pfaar O Demoly P Gerth van Wijk R Bonini S Bousquet J Canonica GW Recommendations for the standardization of clinical outcomes used in allergen immunotherapy trials for allergic rhinoconjunctivitis: an EAACI position paper. *Allergy.* (2014) 69:854–67. 10.1111/all.12383 24761804

[35] Pfaar O Bachert C Kuna P Panzner P Džupinová M Klimek L Sublingual allergen immunotherapy with a liquid birch pollen product in patients with seasonal allergic rhinoconjunctivitis with or without asthma. *J Allergy ClinImmunol.* (2019) 143:970–7. 10.1016/j.jaci.2018.11.018 30508538

[36] Juniper EF Thompson AK Ferrie PJ Roberts JN. Validation of the standardized version of the rhinoconjunctivitis quality of life questionnaire. *J Allergy ClinImmunol.* (1999) 104(2 Pt 1):364–9. 10.1016/S0091-6749(99)70380-5 10452758

[37] Juniper EF Guyatt GH Griffith LE Ferrie PJ. Interpretation of rhinoconjunctivitis quality of life questionnaire data. *J Allergy ClinImmunol.* (1996) 98:843–5. 10.1016/S0091-6749(96)70135-5 8876562

[38] Chen Q Zhang Q Jiang L Li X Liu Y Xie Y Effectiveness of strengthened stimulation during acupuncture for the treatment of allergic rhinitis: study protocol for a randomized controlled trial. *Trials.* (2014) 15:301. 10.1186/1745-6215-15-301 25059460 PMC4133069

